# Applications of Graphene-Based Materials in Sensors: A Review

**DOI:** 10.3390/mi13020184

**Published:** 2022-01-26

**Authors:** Jihong Liu, Siyu Bao, Xinzhe Wang

**Affiliations:** College of Information Science and Engineering, Northeastern University, Shenyang 110819, China; 20184229@stu.neu.edu.cn (S.B.); 20184225@stu.neu.edu.cn (X.W.)

**Keywords:** graphene materials, graphene sensor, biosensors, flexible pressure sensor, photoelectrochemical sensors

## Abstract

With the research and the development of graphene-based materials, new sensors based on graphene compound materials are of great significance to scientific research and the consumer market. However, in the past ten years, due to the requirements of sensor accuracy, reliability, and durability, the development of new graphene sensors still faces many challenges in the future. Due to the special structure of graphene, the obtained characteristics can meet the requirements of high-performance sensors. Therefore, graphene materials have been applied in many innovative sensor materials in recent years. This paper introduces the important role and specific examples of sensors based on graphene and its base materials in biomedicine, photoelectrochemistry, flexible pressure, and other fields in recent years, and it puts forward the difficulties encountered in the application of graphene materials in sensors. Finally, the development direction of graphene sensors has been prospected. For the past two years of the COVID-19 epidemic, the detection of the virus sensor has been investigated. These new graphene sensors can complete signal detection based on accuracy and reliability, which provides a reference for researchers to select and manufacture sensor materials.

## 1. Introduction

A sensor is a kind of detection device that can convert the received information into an electrical signal or other signal output. In this era of intelligence, digitization, and networking, sensors, as “electrical facial features”, have become the main way and means of obtaining information. The requirements for sensor sensitivity and application range are also higher and higher. The sensitive element of the sensor is very important for the performance of the sensor, which directly affects the accuracy, sensitivity, and detection range of the sensor. The applications of sensors in biomedicine, flexible pressure, and photoelectrochemistry introduced in this paper have penetrated all aspects of our life and made indisputable contributions to the development of modernization.

Graphene is a single-layer planar film with a hexagonal honeycomb lattice composed of carbon atoms. Graphene material has excellent electrical and mechanical properties due to its special structure, which has attracted extensive attention in the engineering field. The sensor is considered to be one of the most promising applications of graphene [1]. At present, several methods have been developed to obtain high-quality graphene films, such as the chemical stripping of graphite through compounds, chemical vapor deposition (CVD) on different substrates, mechanical cracking of graphite crystals, and other chemical synthesis methods [2]. Among them, the chemical vapor method is a low-cost, high-quality, and large-scale production method, which plays an important role in various fields [3]. Since graphene is difficult to produce a product as a single raw material, graphene composites are formed by using the outstanding characteristics of graphene and other materials. The size, number of layers, shape, and chemical groups of graphene have a significant impact on the performance of the sensor [4,5]. Graphene-based composites can improve the sensitivity and flexibility of traditional sensors and shorten the reaction time. There is no doubt about the role of graphene in sensors, but how to select appropriate graphene-based materials according to their applications has become a problem [2].

Carbon nanotubes and graphene belong to graphite materials. Structurally, carbon nanotubes are helical, while graphene is a flake, but they all have some common characteristics of graphite, such as excellent conductivity. Therefore, due to the small size, large specific area, different bond states on the surface and inside the particles, and incomplete coordination of surface atoms, carbon nanotubes have also become an ideal additive for conductive materials. As an electrode material, carbon nanotubes have better electrochemical properties than traditional carbon electrodes and can be used to detect a variety of gases such as NO_2_ and NH_3_. Although carbon nanotubes as electrochemical sensors are still in their infancy, they have also shown great potential [6]. After chemical modification, carbon nanotubes can also be used as biomolecular sensors with high sensitivity and selectivity [7]. The vibration characteristics of carbon nanotubes are very important. When carbon nanotubes are used in sensors and other fields, the vibration characteristics determine the working state and performance of the sensor [8]; The deformability and transmittance of carbon nanotube reinforced materials also depend on the vibration characteristics [8]. Therefore, studying the oscillation of carbon nanotubes is conducive to researchers to better understand the mechanical properties of carbon nanotubes and explore the application fields of carbon nanotubes [9]. The author believes that although carbon nano and graphene have similar predecessors, they may have different futures. The application of carbon nanotubes has encountered a bottleneck due to the limitation of the macro size of carbon nanotubes, which cannot be synthesized by the current technology. As a result of its two-dimensional crystal structure, graphene can grow in a large area and has a bright application prospect.

Graphene is a single atom thick honeycomb structure formed by a two-dimensional carbon atom monolayer, so it forms a large surface volume ratio, making it suitable for ultrasensitive detection applications [10,11,12,13,14,15]. In addition, due to its p-orbital electrons, graphene forms π bonds with the surrounding atoms, and the electrons of these π bonds have high sensitivity to any environmental change. Therefore, graphene materials are very suitable for chemical and biosensor applications [10,13,16]. This paper introduces the role of graphene-based sensors in biomedicine, flexible pressure, and photoelectrochemistry, and it lists several specific application examples. Since the preparation of graphene material is difficult, a simple and practical method for preparing graphene biosensors is proposed in this paper. Finally, the applications of graphene materials in biosensors, physical sensors, and chemical sensors are introduced, and the role of graphene materials in sensors is summarized. The difficulties in the development of graphene materials are put forward, and the future development trend of graphene sensors is prospected, which provides a certain basis and reference for the research of graphene sensors.

## 2. Application of Graphene Materials in Biosensors 

The fundamental reason for graphene’s conductivity is that it has unbound free electrons (π electrons) [17]. The biosensor is an instrument that is sensitive to biological substances and converts their concentration into electrical signals for detection. Biosensors provide unprecedented technical support for the detection of some components in modern medicine and the early detection of diseases. Referring to the application of graphene in biosensors in the recent ten years, we found that graphene materials can usually provide higher sensitivity and reduce reaction time for existing biological detection technologies due to their strong adsorption capacity and excellent conductivity [18,19]. Then, we will give several specific examples to illustrate that the addition of graphene can improve the performance of biosensors.

**Figure 1:**
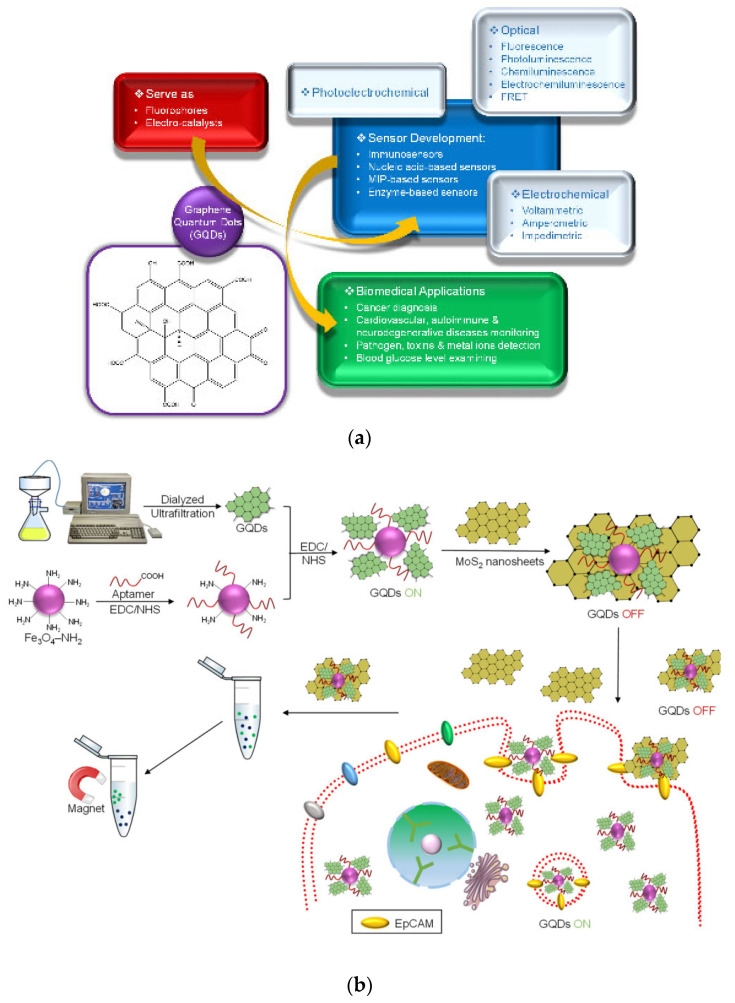

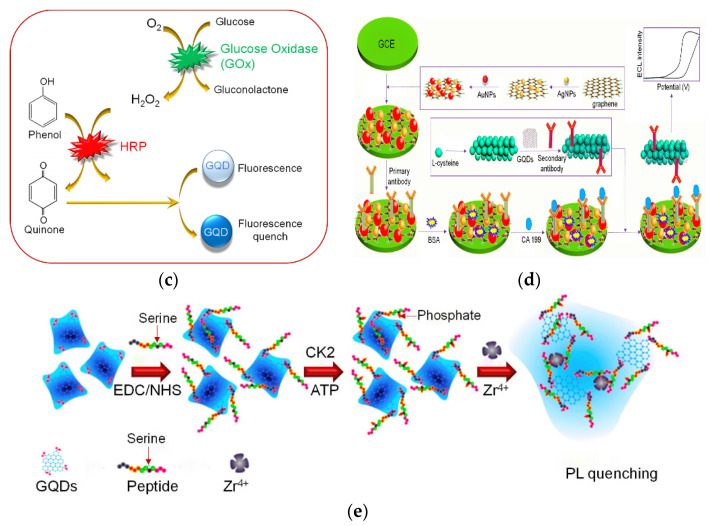
Graphene biosensor. (**a**) Structure of graphene quantum dots and its application in biomedical sensors [18]; (**b**) Sensor for detecting tumor cells based on aptamer/Fe3O4/GQDS/MoS2 [18,27]; (**c**) GQD biosensor based on the synergistic effect of enzyme coupling technology and fluorescence quenching is used to monitor the glucose level in human serum samples [18,28]; (**d**) Sensor for detecting carbohydrate antigen in human serum based on GQDs [18,29]; (**e**) A GQD sensor based on PL for screening protein kinase activity [18,30].

In the past few years, a large number of nanomaterials have been used as signal amplification species [18]. Graphene quantum dots (GQD) have attracted much attention because of their good biocompatibility, catalytic ability, and ability to detect several biomolecules at the same time [18]. The morphological characteristics of GQD are similar to carbon dots and graphene, but there are single or multiple panels on its side, which look spherical or spherical and can provide a large number of positions for biomolecules [20,21]. Figure 1a shows the chemical structure of graphene quantum dots and their application in biomedical sensors. Although the semiconductor quantum dots in the market are developing ideally due to their good stability and stability, most of them have heavy metals, which hinders their biomedical applicability [22,23,24]. Graphene quantum dots can be used as an electrocatalyst and nano enzyme to determine the target analyte without a label [18,25]; graphene quantum dots can be fluorescent-labeled as attractive fluorescent groups [18,26]; Figure 1b–e will show examples of several specific biomedical graphene quantum dot sensors.

Molecularly imprinted polymer (MIP) is a promising technology. The GSCR-MIP sensor can be used to detect amino acids for the early detection of cancer diseases in the blood [31]. High conductivity is very important for MIP because it will directly affect the electron mobility of the polymer itself, thus affecting the sensitivity and response time of sensing materials [31,32,33]. The researchers found that graphene sheet/Congo red (GSCR) has excellent conductivity even when the detected amino acid concentration is very low [34]. Oxidized grain in GSCR-MIP was synthesized by the Hummer method [35]. Add 5.0 mg Congo red into 40.0 ml of graphene solution, stir, filter, and dry to prepare the GSCR-MIP composite [36]. It can be seen from the experimental results of relevant articles that when detecting serine in the sample, the improved sensor with graphene material is added, and the leakage current is increased compared with the improved sensor [31]. This shows that the use of SCR material can improve the conductivity of the sensor and improve the performance of the sensor [31].

Another graphene biosensor to be introduced is the SPR (Surface Plasmon Resonance) sensor. The SPR sensor has the ability of real-time, accurate, and minimally invasive concentration analysis, label-free, high-precision detection, and monitoring [37,38]. The performance of an optical fiber SPR sensor (FO-SPR) with graphene material is better than that of a traditional prism SPR sensor [39]. The FO-SPR sensor can be used for the early detection of breast cancer and other medical processes [37,38,40]. The main performance parameters of the SPR sensor are sensitivity (SN) and detection accuracy (SNR) [39]. The research shows that by adding protective and absorbent graphene layers to the metal layer, the sensitivity of the FO-SPR sensor can be improved by about 50% [39,41]. This is due to the high conductivity, thermal conductivity, and biocompatibility of graphene [42]. Therefore, for the FO-SPR sensor, coating graphene protection and the absorption layer on the metal layer can improve the service life, sensitivity, and detection accuracy of the SPR sensor [38].

Graphene is considered to be a promising material due to its excellent electrical conductivity [43]. Some researchers believe that graphene will replace silicon in the future [44], but the biggest challenge in this process is due to the complex manufacturing technology and the high production cost of graphene-based devices [43]. Among many methods for preparing graphene sensors, CVD technology can reliably and effectively produce graphene on a large scale [45]; however, the graphene devices obtained by this method will reduce the quality of graphene [46], and there will be residues that are difficult to remove [47]. When making graphene sensors, some researchers used a new preparation method to avoid the difficulty of graphene preparation, that is, a new method of making graphene sensors in the PDMS channel, but this method is only suitable for making biosensors. In this method, the copper plate and graphene are connected to the PDMS surface by mechanical pressure, and then, the PDMS layer is bonded to another PDMS substrate containing microchannels. A graphene sensor is obtained on PDMS, while copper is etched by etchant through the microchannel. The method is simple, easy to use, and scalable. Graphene sensors for biomedical applications can be obtained in microchannels [43].

## 3. Application of Graphene in Physical Sensors

An important type of physical sensor is the pressure sensor. Graphene material has important applications in pressure sensors, and with the development of intelligence, flexible pressure sensors have attracted much attention. As a new type of electronic product, the flexible pressure sensor has better application prospects in the fields of biomedicine, robot touch, and human–computer interaction than ordinary rigid sensors. The flexible pressure sensor is becoming more and more important; it is the main way of modern electronic products input and control [48,49]. At the same time, a large area tactile sensor can be applied to the skin of the robot. This composite material can sense the touch in the range of 0.02 to 10 N, which belongs to the category of human tactile perception [48]. In addition, in the past decade, the demand for flexible wearable sensors has soared. Due to the dispersion, high specific surface area volume ratio, and excellent physical and chemical properties of one-dimensional and two-dimensional nanomaterials, they can be used to develop high sensitivity and scalable tactile sensing systems [48,50]. More importantly, compared with traditional films, inorganic nanostructures are easier to combine with flexible materials [51]. Nanomaterials, carbon nanofibers, carbon black, and graphene are often combined with elastomers such as polydimethylsiloxane (PDMS), eco flex, and rubber to make polymer composite sensors [52]. Based on this premise, we found that graphene is a two-dimensional nanostructured material that can be synthesized in a large area [48,53,54]. Graphene-based pressure sensors have attracted much attention due to their high sensitivity and large area scalability [48].

The research of flexible pressure sensors mainly changes the microstructure of the conductive layer of the sensor to improve the sensing performance of the sensor [52,55]. The following will introduce specific examples of several graphene flexible pressure sensors to reflect the role of graphene materials in flexible pressure sensors.

**Figure 2:**
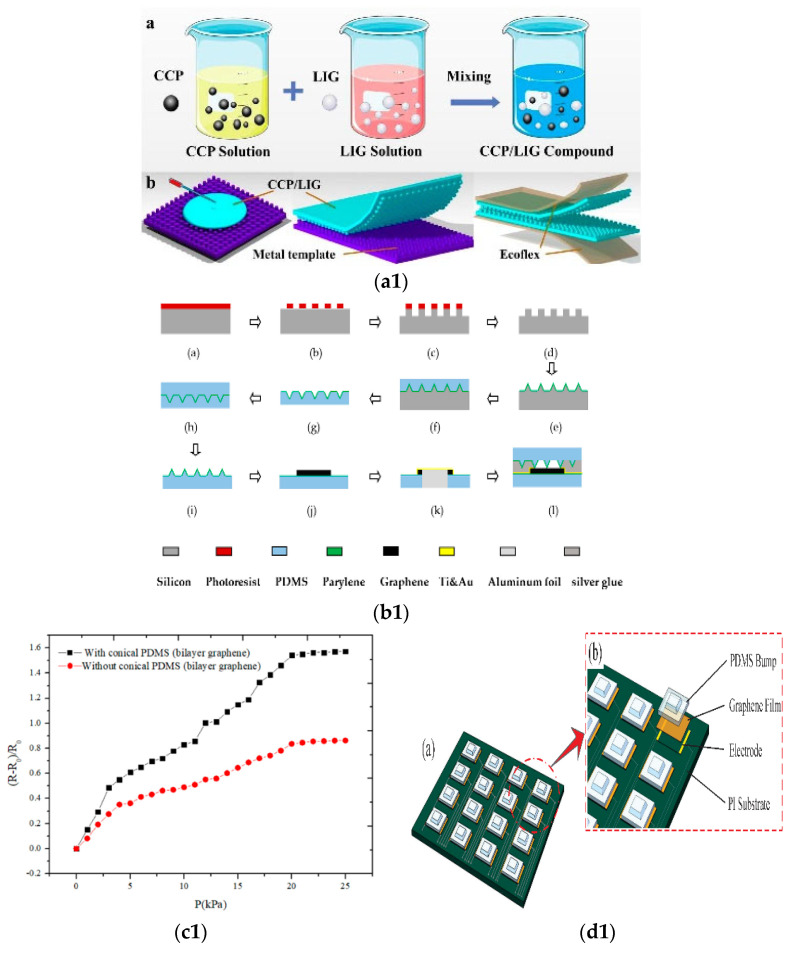

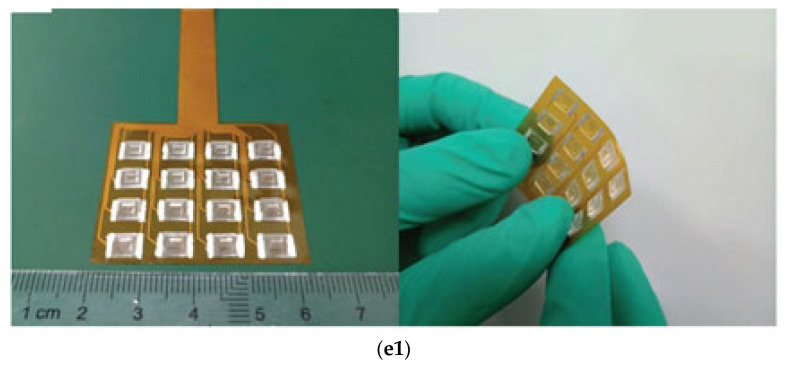
Flexible pressure sensor. (**a1**) Manufacturing process diagram of LIG/CCG sensor [52]; (**b1**) Preparation process of a sensor made of conical microstructure PDMS substrate and double-layer graphene [58]; (**c1**) Sensitivity curve of the sensor with and without conical microstructure [58]; (**d1**) Structure and enlarged details of electronic skin [59]; (**e1**) Size and photos of electronic skin [59].

Due to the rapid development of laser direct writing technology, more researchers use laser-assisted technology to obtain graphene on flexible carbon substrate [52]. Among various high-performance composite graphene materials, laser-induced graphene (LIG) has been widely used in the field of flexible pressure sensors in recent years because of its simple preparation, low cost, and large area [52,56]. Some researchers mixed LIG and conductive carbon slurry to prepare the conductive mixture and made a pressure sensor by laser processing technology. The manufacturing process is shown in Figure 2(a1). Then, by changing the ratio of laser flux to LIG in the composite, the sensitivity of the LIG/CCP sensor with a bionic structure is analyzed to determine the optimal laser flux and LIG mass fraction. The analysis of experimental results shows that LIG nanostructures have the function of repairing conductive network defects and can improve the sensitivity of the sensor in a certain range [52,57].

Some researchers improved the sensitivity based on a wide detection range and designed a PDMS double-layer graphene flexible pressure sensor with a conical microstructure [58]. The designer combines the conical microstructure PDMS substrate prepared by various processes with double-layer graphene to package the flexible pressure sensor [58]. The preparation process is shown in Figure 2(b1). The double-layer graphene material makes the resistance of the sensor change significantly even under slight pressure. The conical microstructure PDMS substrate can improve the sensitivity of the sensor even when the deformation of the double-layer graphene sensor reaches saturation [58], as shown in the graph in Figure 2(c1). As a result of its high sensitivity and flexibility, the sensor can be applied to the real-time monitoring of human physiological signals.

Another researcher developed a flexible electronic skin based on graphene film. This electronic skin is an array of pressure sensors. The picture of the sensor is shown in Figure 2(d1,e1). The principle is that the piezoresistive effect of graphene material is used. When under pressure, the C-C bond will differentiate or break, resulting in the change of resistivity of graphene film [59,60]. Since graphene film has excellent sensitivity and flexibility, the sensor has high sensitivity [59].

These experimental results show that graphene material has good flexibility, conductivity, and sensitivity, and it can realize the function of the pressure sensor. It provides ideas and methods for making flexible pressure sensors in the future and has development potential.

The application of graphene in flexible pressure sensors involves many other fields. For example, a flexible dual-mode sensing system is designed to synchronously monitor the pressure and inertia information of finger movement [61]; A wearable elastic dry electrode that can be used to monitor the subtle changes of ECG information in real-time is designed by using a graphene sponge [62]. These applications are based on the flexibility, wearability, and biocompatibility of graphene materials. To compete with the current traditional pressure sensors, graphene pressure sensors that are compatible with temperature-sensitive substrates, easier to manufacture, and more sensitive need to be developed [63].

## 4. Application of Graphene in Chemical Sensors

Graphene is a kind of new material with a single-layer two-dimensional lattice structure, which is closely packed by SP2 hybrid-connected carbon atoms [64]. The original graphene is chemically inert and is not easy to react with other substances [65]. However, stable polymers can be formed by breaking covalent bonds and combining them with organic compounds [65]. These graphene-doped polymers have good conductivity and optical properties, and graphene-based chemical sensors have better detection and conductivity than traditional gas and ion sensors. In this section, several photoelectric sensors with good performance in recent years are reviewed, which shows that the performance of chemical sensors doped with graphene has been greatly improved.

There is a metal-coated graphene optical fiber probe sensor for nitrate sensor [66]. The tip of the standard single-mode fiber was cut, graphene was coated by impregnation, dried for 1 h, and used as the contact end of the sensor [66]. In the experiment, the original optical fiber probe sensor and graphene-coated optical fiber probe sensor were inserted into the same concentration of nitrate solution, respectively. During the experiment, the nitrate concentration increased from 0 to 50 ppm. The experimental results show that the optical fiber probe sensor with a graphene layer has a greater difference in light intensity at different concentrations; that is, the sensor covered with graphene has higher sensitivity.

In recent years, a new type of composite sensor has emerged, that is, the PPy layer is electrodeposited on CVD-grown graphene. This composite sensor has good performance in ammonia monitoring and sensing [65]. First, electrodes were deposited during emission, then graphene was added, and finally, polypyrrole was synthesized [65]. During the experiment, ammonia (NH_3_) was obtained by diluting 100 ppm NH_3_ in wet air at 20 °C [65]. The resistance between the two electrodes ΔR/R0 = (RS- R0)/R0(%) is used to detect the performance of the sensor [65]. From the experimental results, we can conclude that the new graphene polymerization hybrid sensor has higher sensitivity to ammonia (NH_3_), lower production cost, and better structural stability and durability than the traditional metal oxide or catalytic metal ammonia sensor (NH_3_) [65].

**Figure 3:**
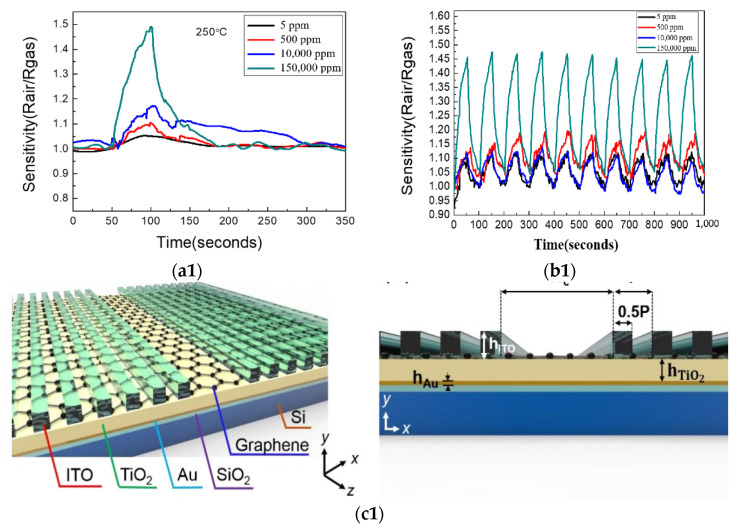

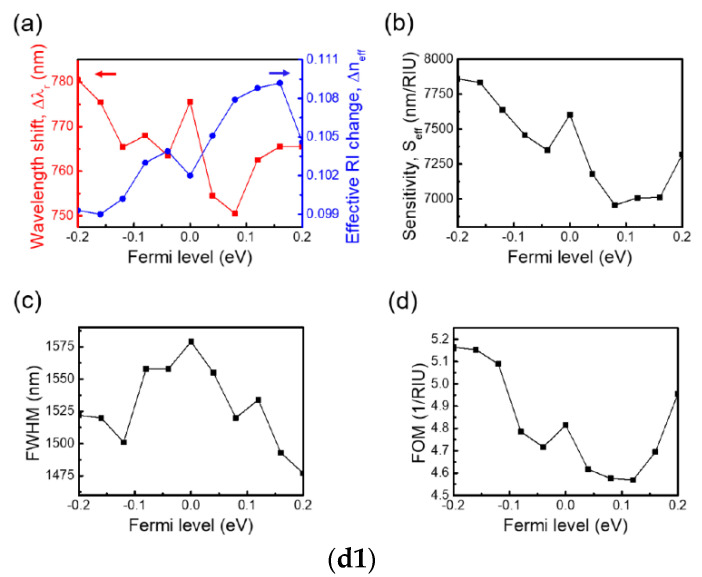
Photoelectrochemical sensor. (**a1**) Sensitivity curves of ZnO/graphene sensor under different concentrations of hydrogen [67]; (**b1**) Reproducibility experiment of ZnO/graphene sensor [67]; (**c1**) Structure diagram and cross-section diagram of resonant cavity plasma photon biochemical sensor [68]; (**d1**) Effect of adjusting Fermi level of graphene on the sensitivity and wavelength of the sensor [68].

Some researchers prepared graphene films by chemical vapor deposition and transferred them to the silicon substrate by PMMA transfer technology. One-dimensional ZnO nanostructures were prepared and grown on the top surface of graphene. The graphene/ZnO nanostructured gas sensor has good sensitivity and repeatability for hydrogen in a certain range. This is attributed to the high specific area of nano ZnO and the p-n heterojunction formed between ZnO and graphene [67]. As shown in Figure 3(a1,b1), the ZnO/graphene sensor has high sensitivity and repeatability and can be used for hydrogen detection.

Applying different bias voltages to graphene can change its Fermi level, so the optical properties of the resonator will also change. Some researchers use this adjustable property of graphene to improve the performance of the sensor and change the resonance wavelength to study fingerprint detection [68]. The resonant cavity plasma photon biochemical sensor proposed in this paper is shown in Figure 3(c1). The influence of adjusting the Fermi level of graphene on the sensitivity and wavelength of the sensor is shown in Figure 3(d1).

## 5. Challenges and Prospect

This paper summarizes the important role of graphene in biosensors, physical sensors, and chemical sensors, and it lists several application examples, which provides a basis for researchers to develop sensor materials in corresponding fields. From the above introduction, we can see that graphene has become an ideal sensor material due to its superior conductivity, biocompatibility, and uniform distribution of active groups [69].

The role of graphene in sensor materials can be summarized as follows:

(a) Improve the sensitivity of the sensor and shorten the response time;
(b) Combined with flexible materials, it can be extended to a large area.

However, most graphene-based sensors are still in the experimental stage and have not been commercialized. The main reason is that the preparation of graphene film cannot achieve large-scale and high-quality production. At present, the main preparation methods of graphene films and their advantages and disadvantages are shown in Table 1. From the table, we can see that the chemical vapor deposition method and redox method with copper foil as substrate are more suitable methods to realize large-scale production. In the last two or three years, researchers have also optimized the production quality of graphene by changing the production method or different reducing agents according to different application environments and the principles of these two production methods. For example, when preparing graphene material by the CVD method, the copper surface suitable for single-layer graphene synthesis was found by changing the purity of copper foil [70]. When making a non-enzymatic sensor for glucose detection, a novel and efficient hydrothermal method was used to reduce the coincidence material containing graphene oxide [71].

**Figure 4:**
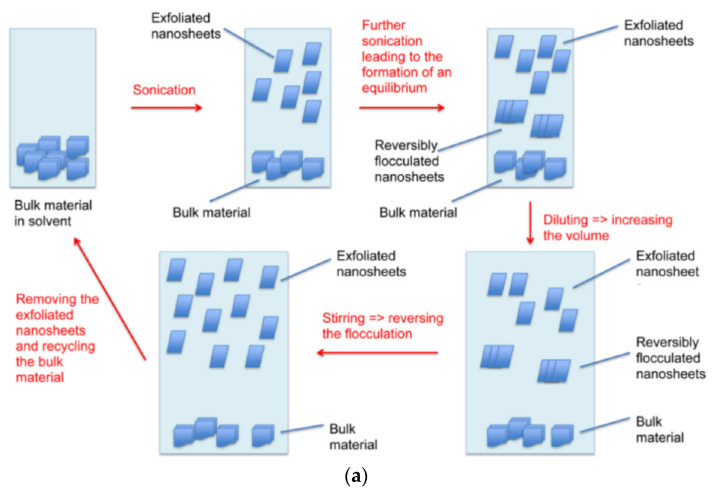

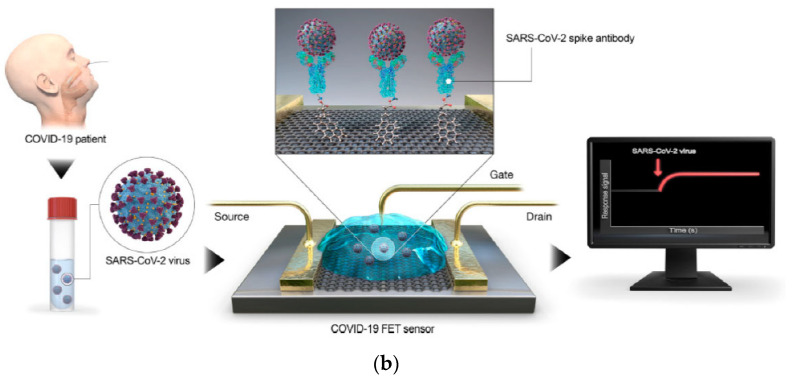
(**a**) In the improved LPE method, when the sample reaches equilibrium to produce flocculated nanosheets, dilute and stir the sample, remove the exfoliated 2D materials, recover the bulk materials, and repeat the process [72]. (**b**) 2019 coronavirus disease (COVID-19) field effect transistor (FET)—sensor. Using graphene as sensing material, 1-pyrene butyrate two imide ester (PBASE) was used as an interface molecule and probe connector, and a SARS-CoV-2 peak antibody was used to modify graphene [75,78].

Liquid phase stripping (LPE) is also a widely used two-dimensional material preparation method. Although this method has great potential, the yield is still not high. When studying the changes in ultrasonic-assisted LPE, some researchers found that the low yield was due to the balance between the angular removal of nanosheets and flocculation of nanosheets during ultrasonic treatment [72]. Finding the reasons for low production and avoiding this balance in future production is of great significance for the production and commercialization of 2D materials (including graphene materials). Finally, the experimenters also showed that the repeated LPE process can improve the yield, as shown in Figure 4a. Compared with traditional semiconductor materials, the synthesis of graphene quantum dots is underdeveloped with the increase in size [2,73], and the attraction between graphene increases, resulting in the decrease in solution solubility, which will seriously limit the size of the stable colloidal graphene structure and hinder the research of the quantum content system [2,74], which is also the reason why graphene cannot be produced in high quality and large scale at present. From the above analysis, it can be seen that graphene has a promising future in the application field of sensors, but solving the manufacturing problem of graphene material is the key. While researchers continue to find the excellent performance of graphene in the sensor field, they should also develop a way to produce this sensor in large quantities and high quality according to the basic principle of production to bring the sensor with excellent performance to the market. In addition, when a graphene sensor is applied to human monitoring, we should pay attention to whether the sensor material has an impact on human safety [2].

At present, the graphene sensor for COVID-19 detection has also been developed. It will detect viruses faster and more accurately, which will provide a great convenience for epidemic prevention and control. Graphene has great potential in liquid biopsy biosensors because of its excellent properties. The development of diagnostic technology is very important for the management of the COVID-19 epidemic. Efforts are being made to improve the detection of COVID-19, and a variety of targeted biosensors [75,76,77] have been developed. For example, LSPR can detect respiratory samples employing a biosensor. A biosensor based on field-effect transistor (FET) is used to detect spike protein on COVID-19 [75,78]. The detection principle of the sensor is shown in Figure 4b. Although graphene-based sensors for the detection of COVID-19 are not popular, graphene materials will play a leading role in virus detection. We hope that with the joint efforts of chemists, physicists, and material scientists, graphene can show more characteristics in future science and technology and contribute to human life in the future.

**Table 1:** Methods, advantages, and disadvantages of making graphene materials.

Preparation Method	Basic Principles	Characteristic
micromechanical exfoliation	The mechanical force is used to overcome the van der Waals force between graphene and separate graphene [79].	Single graphene was obtained for the first time, and the process is complex, which cannot realize large-scale production [80].
chemical vapor deposition	Pyrolytic carbon was formed on the surface of the metal substrate with a carbon compound as a precursor. By controlling the reaction conditions, the pyrolytic carbon forms graphene through nucleation and repolymerization [80].	A large area and high-quality graphene can be prepared by using copper foil as a substrate [81,82].
epitaxial growth	The crystal structure is grown from another crystal structure using lattice matching [80].	One or two layers of graphene can be obtained under harsh preparation conditions [80].
the reduction of graphene oxide (GO) solution	Graphene was obtained by the reduction of graphite oxide [83].	With low cost and high yield, it can be produced on a large scale [80].

## 6. Conclusions

This paper introduces the basic structure information and characteristics of graphene materials, expounds on the role of graphene materials in biomedical sensors, flexible pressure sensors, and photoelectrochemical sensors, and lists application examples in various fields. Finally, various preparation methods of graphene materials are compared, the difficulties encountered in the commercialization of graphene sensors are put forward, and some suggestions for the future development of graphene sensors are given.
